# Dabigatran in patients with atrial fibrillation: perioperative and periinterventional management

**DOI:** 10.1007/s00508-012-0166-5

**Published:** 2012-05-11

**Authors:** A. Weltermann, M. Brodmann, H. Domanovits, B. Eber, M. Gottsauner-Wolf, W. M. Halbmayer, J. M. Hiesmayr, P. A. Kyrle, F. Längle, F. X. Roithinger, H. Watzke, R. Windhager, C. Wolf, R. Zweiker

**Affiliations:** 1 Department of Medicine I, Elisabethinen Hospital Linz, Fadinger Straße 1, 4020 Linz, Austria; 2 Department of Internal Medicine, Division of Angiology, Medical University Graz, Graz, Austria; 3 Department of Emergency Medicine, Medical University Vienna, Vienna, Austria; 4 Department of Internal Medicine II, Division of Cardiology, Clinic Wels-Grieskirchen, Wels, Austria; 5 Department of Internal Medicine II, Division of Cardiology, Medical University Vienna, Vienna, Austria; 6 Institute of Laboratory Medicine, Municipal Hospital Hietzing-Rosenhügel, Vienna, Austria; 7 Department of Cardiothoracic and Vascular Anaesthesia and Critical Care Medicine, Medical University Vienna, Vienna, Austria; 8 Department of Internal Medicine I, Medical University Vienna, Vienna, Austria; 9 Department of Surgery, Landesklinikum Wiener Neustadt, Wiener Neustadt, Austria; 10 Department of Internal Medicine, Landesklinikum Thermenregion Mödling, Mödling, Austria; 11 Department of Orthopaedics, Medical University of Vienna, Vienna, Austria; 12 Department of Cardiology, Sozialmedizinisches Zentrum Ost (Donauspital), Vienna, Austria; 13 Department of Internal Medicine, Division of Cardiology, Medical University Graz, Graz, Austria

**Keywords:** Dabigatran, Bridging, Surgery, Endoscopy, Bleeding, Dabigatran, Bridging, Chirurgie, Endoskopie, Blutung

## Abstract

In any type of invasive surgery, the patient’s individual risk of thromboembolism has to be weighed against the risk of bleeding. Based on various everyday situations in clinical routine, the purpose of the present expert recommendations is to provide appropriate perioperative and periinterventional management for patients with atrial fibrillation undergoing long-term treatment with the thrombin inhibitor dabigatran. As we currently have no routine laboratory test to measure therapeutic levels of the substance or the risk of bleeding, general measures such as a standardized documentation of the patient’s history, a sufficient time interval between the last preoperative dose and the procedure, and careful control of local hemostasis should be given special attention.

## Abstract (de)

Chirurgische und andere interventionelle Eingriffe erfordern ein individuelles Abwägen des Blutungsrisikos gegenüber dem Thromboserisiko des Patienten. Da sich die neuen Antikoagulantien von den klassischen Vitamin K Antagonisten und Bezug auf Halbwertzeit und Laborkontrollen deutlich unterscheiden, sind für diese Substanzen andere Vorgehensweisen zu definieren. Ziel der vorliegenden Expertenempfehlung ist es, ein optimales perioperatives/interventionelles Management für Patienten zu beschreiben, die eine Dauertherapie mit dem kürzlich zugelassenen oralen Thrombininhibitor Dabigatran erhalten. Anhand verschiedener klinischer Routinesituationen wird das Vorgehen erläutert und der Evidenzgrad der Empfehlung angegeben. Da ein Routine-Labortest zur Bestimmung des Wirkspiegels nicht überall verfügbar ist, ist auf eine sorgfältige Anamnese, einen ausreichenden Zeitabstand zwischen der letzten präoperativen Gabe von Dabigatran und dem geplanten Eingriff sowie einer sorgfältigen Blutstillung zu achten.

## Background

In any type of invasive surgery, the patient’s individual risk of thromboembolism has to be weighed against the risk of bleeding (and its potential sequelae). This is especially true for patients who receive anticoagulation therapy. This issue has been the subject of numerous clinical studies, which formed the basis for the recommendations concerning perioperative use of vitamin K antagonists (VKA) and low-molecular-weight heparins (LMWH).

Analogous to the intake of the currently available anticoagulants, it may be assumed that new anticoagulants such as dabigatran (a direct thrombin inhibitor for oral administration) lead to a higher risk of bleeding during surgical or invasive procedures, and following acute injury [1]. Systematic investigations demonstrating the association between dosage, the interval between the last preoperative dose and clinical progress (risk of bleeding and thromboembolism) after surgical procedures are not available for dabigatran. The RE-LY^®^ study protocol provided no specific instructions as regards periinterventional bridging. Clinical data from the RE-LY^®^ study are only available for those patients who underwent electrical cardioversion during the observation period. The European as well as the U.S. American Summary of Product Characteristics contain basic information about bridging, but specific recommendations for the various surgical and interventional procedures are not provided.

The purpose of the present recommendations is to serve as a supplement to the Summary of Product Characteristics and assist the physician in clinical routine by way of enabling him/her to provide appropriate perioperative and periinterventional management for patients with atrial fibrillation undergoing long-term treatment with dabigatran. As we currently have no routine laboratory test to measure therapeutic levels of the substance or the risk of bleeding, general measures such as a standardized documentation of the patient’s history, a sufficient time interval between the last preoperative dose and the procedure, and careful control of local hemostasis should be given special attention. One should keep in mind, that the concomitant use of acetylsalicylic acid (ASA), clopidogrel or nonsteroidal antiinflammatory drug (NSAID) is associated with an increased bleeding risk. Based on various everyday situations in clinical routine, optional approaches to periinterventional bridging will be described in the following. The recommendations concerning bridging with dabigatran, especially the reinstitution of anticoagulation after an intervention, refer to an uneventful postoperative or postinterventional course. In cases of complications—especially bleeding—the clinician should select an individually adapted therapeutic approach.

## Methods

The existing recommendations of the consensus group were formulated on the basis of the following sources of data:

1. Recommendation for the management of acute or elective interventions in accordance with the protocol of the RE-LY^®^ study.
2. Results of own literature research and evaluation, especially studies concerning dabigatran (e.g., published pharmacological data).
3. Summary of Product Characteristics of the European Medical Association EMA, U.S. Food and Drug Administration USA (FDA).
4. International recommendations on the management of currently available anticoagulants.

All recommendations were adopted in “strong” consensus, i.e., more than 95 % of the participants expressed their approval. While every care has been taken, the consensus group can take no responsibility for the accuracy of the data—especially those concerning dosage. The strength of the recommendation is based on the classification used to formulate the guidelines of the European Society of Cardiology (Table 1) [2].

**Table 1:** The strength of the expert recommendation is based on the classification used to formulate the guidelines of the European Society of Cardiology [1]

*Class of recommendation*	
Class 1	Evidence and/or general agreement that a specific medication or intervention is beneficial, useful, and effective
Class 2	Nonconcurrent evidence and/or no concurrence that a specific medication or intervention is beneficial, useful, and effective
Class 3	Evidence and/or general agreement that a specific medication or intervention is not beneficial, useful, or effective. Therefore, the medication/measure is not recommended
*Level of evidence*	
Level A	Study results from several randomized clinical studies or metaanalyses
Level B	Study results of a randomized clinical study or large nonrandomized studies
Level C	Consensus opinion of the expert group and/or small studies, retrospective studies, register data

### Pharmacokinetics and effects on coagulation assays

Dabigatran etexilate may be taken independent of the ingestion of food [3–5]. The capsule should be swallowed whole. Opening the capsule and administering its contents (e.g., when the substance is administered via a PEG tube) is not advisable because the bioavailability of the substance may increase by up to 75 % [5]. The maximal effect of dabigatran (active substance) occurs as early as 1–2 h after its oral intake [3–5]. With twice daily dosing, the maximum concentration in plasma (Cmax) at steady state is about 30 % higher than after the first dose [5]. The half-life of dabigatran is longer than that of LMWH and dependent on renal function (creatinine clearance > 80 ml/min (normal): t_½_ 13 h; creatinine clearance 30–50 ml/min: t_½_ 18 h) [3–8]. However, the half-life may be as long as 21.6 h even in case of normal creatinine clearance (interindividual variability approximately 26 %) [5]. Therefore, prior to a surgical procedure with a higher risk of bleeding, the clinician is advised to maintain a longer safety interval as compared with LMWH (see details below).

As dabigatran may influence several coagulation tests, the clinician is likely to obtain “unusual” findings on routine coagulation tests during regular intake of dabigatran (see below). Potential changes in routine coagulation parameters due to dabigatran are summarized in Table 2 [8,9]. However, normal findings on coagulation monitoring do not rule out a significant effect of dabigatran. In case attempts are made to perform routine diagnostic procedures for determination of the patient’s coagulation preoperatively, these should be performed during trough levels, i.e., prior to the next scheduled intake of dabigatran (**Recommendation 1C**) [9]. If dabigatran has to be paused during the intervention, the preoperative diagnostic procedure for coagulation monitoring should be performed shortly before the operation and after an appropriately long interruption of treatment with dabigatran (optimally ³ 24 h;**Recommendation 1C**).

**Table 2:** A summary of the systematic evaluation reveals the following potential changes in routine coagulation parameters due to dabigatran. (Adapted from [7])

Parameter (unit)	Change (dose dependent)
Prothrombin time (PT) (s)	­
International normalized ratio (INR)	­
Partial thromboplastin time (aPTT) (s)	­­
Thrombin time (TT) (s)	­­­ (to nondetectability)
Activated clotting time (ACT) (s)	­­
Fibrinogen assay (Clauss’ method) (mg/dl)	¯¯
Coagulation factors VIII, IX, XI, XII (%)	¯¯
Coagulation factors II, V, VII, X (%)	¯

The following details concerning the individual laboratory tests refer to maximal levels, which may be anticipated about 2–3 h after oral intake.

In the majority of patients the 150 mg BID dose as well as the 110 mg BID dose prolong the*activated partial thromboplastin time* (*aPTT*) [8]. During the intake of twice daily dose of 150 mg dabigatran, the median peak aPTT is twofold that of control. Twelve hours after the last dose, the median aPTT prolongation is increased 1.5-fold. Nevertheless, aPTT should not be used as a measure of the anticoagulant effect in clinical routine because the degree of correlation is only moderate: at the 150 mg BID dose, 15 % of patients demonstrate no or minimal prolongation of aPTT (< 40 s) [8]. During acute interventions it should be noted that as many as 10 % of patients still demonstrate a twofold prolongation of their aPTT 12 h after the last dose as compared with their baseline values [8]. It has to be noted, that the aPTT is not a standardized test. Thus, the results might vary dependent on the aPTT-assay used.

Usually the*International Normalized Ratio* (*INR*) and*Prothrombin Time* (*PT*) are hardly influenced by dabigatran (INR may be normal or slightly increased) [8]. Caution is advised during interpretation of the INR value when switching to Pradaxa or a VKA: the INR value may be falsely high, i.e., it may be higher than it is in case of monotherapy with a VKA. Therefore, the actual anticoagulatory effect of the VKA should be tested again by redetermining INR 48 h after conclusion of dabigatran therapy.

The thrombin time (TT) is too sensitive for monitoring dabigatran [5,8,9]. However, the TT is most useful as a sensitive method for determining if any dabigatran is (still) present. The*Hemoclot test* (Hemoclot^®^ Thrombin Inhibitor Assay, Hyphen BioMed, Neuville-sur-Oise, France) is a diluted TT coagulation assay specifically calibrated to determine the anticoagulatory effect of dabigatran [5,8] In contrast to the TT assay, the Hemoclot test is prolonged in a linear dose-dependent manner. However, the optimal therapeutic range is currently not known. Normalization of the Hemoclot test after discontinuation of dabigatran (e.g., preoperatively) practically rules out a significant anticoagulatory effect. In addition to the Hemoclot test,*Ecarin Clotting Time* (*ECT*) is a sensitive test to determine the anticoagulatory effect of dabigatran, but is performed at just a few centers [8].

The*Activated Clotting Time* (*ACT*) is the most frequently used bedside coagulation test to measure the anticoagulatory effect of unfractionated heparin during cardiac catheterization or any type of cardiac surgery. Dabigatran causes a significant prolongation of ACT in vitro (analogous to aPTT). However, no correlation is noted especially at higher concentrations of dabigatran [8]. ACT measured by rapid thrombelastography (rTEG) may be helpful to prove an anticoagulant effect of dabigatran in trauma patients [1].

### Tooth extractions or other minor surgical procedures

The Summary of Product Characteristics provides no recommendations concerning the management of tooth extractions or minor surgical procedures which permit good control of local hemostasis. International guidelines such as the ACCP guidelines recommend that the intervention be performed under continuous anticoagulation in the therapeutic target range (INR 2.0–3.0) during the intake of VKA, as some studies have demonstrated a higher risk of thromboembolism when the therapy is discontinued and, conversely, the risk of severe bleeding is very low under ongoing anticoagulation [8].

From orthopedic studies with dabigatran in may be concluded that the 110 mg dose initiated shortly postsurgery (1 to 4 h postoperatively) is safe [10,11]. The degree of the risk of bleeding under the 150 mg BID dose and the safety of continuing the preoperative therapy during minor surgery are not known.

Based on extrapolation of the data concerning conventional anticoagulants (VKA, heparin) and the low risk of severe bleeding, the following procedure is recommended:

1. Laboratory testing of dabigatran prior to the intervention is not meaningful (**Recommendation 3C**).
2. Interruption of the treatment with dabigatran is not indicated (**Recommendation 3C**).

### Electrical cardioversion

A subgroup analysis of the RE-LY study showed that electrical cardioversion under dabigatran is associated with similar low rates of complications as those in patients undergoing closely monitored treatment with VKA [12,13]. In total, 1983 electrical cardioversions were performed in 1,270 patients (7 % of the study population): 647 and 672 procedures were performed in patients receiving 110 and 150 mg dabigatran BID, respectively, as compared with 664 in patients receiving warfarin. Prior to cardioversion, a transesophageal echocardiography was performed in 26 % (110 mg dabigatran BID), 24 % (150 mg dabigatran BID), and 13 % (warfarin), of which a thrombus was identified in 1.8, 1.2, and 1.1 %. At 30 days, no significant differences were observed with respect to the rate of stroke or systemic embolism (0.8, 0.3, and 0.6 %) and major bleeding rates (1.7, 0.6, and 0.6 %).

The Summary of Product Characteristics provides the advice that patients can stay on dabigatran etexilate while being converted [5]. This statement constitutes the basis of the following expert recommendation:

1. A laboratory testing of dabigatran prior to electrical cardioversion is not meaningful (**Recommendation 3B**).
2. Interruption of treatment with dabigatran is not indicated (**Recommendation 3B**).

The international recommendations for prevention of systemic embolism (cardioversion for atrial fibrillation < 48 h or after exclusion of a thrombus by transesophageal echocardiography, otherwise no earlier than after 3 weeks of therapeutic anticoagulation) must, of course, be followed.

### Catheter ablation for atrial fibrillation (isolation of the pulmonary vein)

As we have data confirming the safety of ablation of atrial fibrillation under VKA (INR 2.0–3.0) [14], the following procedure may be concluded by analogy and recommended because of the low risk of complications and bleeding:

1. A preinterventional laboratory testing of dabigatran is not meaningful (**Recommendation 3C**).
2. The last dose of dabigatran should be taken the evening before the intervention (**Recommendation 1C**).
3. In patients whose postinterventional course is uneventful, dabigatran should be continued at the usual dose on the evening of the intervention (**Recommendation 1C**).

A potential alternative would be bridging with heparin, which will ensure continued therapeutic anticoagulation in an analogous manner. According to the Summary of Product Characteristics we recommended to wait (at least) 12 h after the last dose before switching from dabigatran etexilate to a parenteral anticoagulant [5].

### Elective cardiac catheterization

Patients with atrial fibrillation have a high risk of cardiovascular complications per se. The ACCP and ESC guidelines provide no recommendations as to how one should deal with VKA in the course of elective cardiac catheterization. During a routine intervention with no additional anticoagulation, as a rule unfractionated heparin at a therapeutic dose is administered. Dabigatran has not been investigated yet in respect of its safety and efficacy for catheter-based interventions. Due to the potential risk of complications and the possible need for cardiac surgery, interruption of treatment with dabigatran is advisable.

Therefore the following is recommended:

1. A preinterventional laboratory testing of dabigatran is not meaningful (**Recommendation 3C**).
2. Dabigatran should be paused the day before the intervention (Day - 1;**Recommendation 1C**). The treatment should be discontinued earlier (at least 2 days) in case of renal failure (creatinine clearance < 50 ml/min;**Recommendation 1C**).
3. The treatment with dabigatran should be continued on the evening of the intervention, but no earlier than 4 h after the last dose of heparin (**Recommendation 1C**).

From the RE-LY study, it was observed that ASA or clopidogrel comedication with dabigatran etexilate at dosages of 110 or 150 mg twice daily may increase the risk of major bleeding. If the patient requires inhibition of platelet function and simultaneous anticoagulation after the intervention, the dose of dabigatran should be reduced during this period from 150 to 110 mg BID (**Recommendation 1C**; conclusion by analogy, based on the national recommendation of VKA therapy aiming at a target INR of 2.0–2.5 during this period and on the Summary of Product Characteristics) [5]. Alternatively, during this interval the patient may be given oral anticoagulation with VKA aimed at a target INR of 2.0–2.5 [15].

### Endoscopic low-risk interventions in the gastrointestinal tract and low-risk vessel punctures

Endoscopic low-risk interventions include gastroscopy or colonoscopy with or without diagnostic biopsies of the mucosa, biliary stent implantation and endosonography (without biopsy). Low-risk vessel punctures include withdrawal of peripheral venous blood and the placement of small-lumen central-venous catheters in vessels, which can be easily compressed from the outside in case of bleeding (jugular vein, femoral vein). No recommendations are provided in the Summary of Product Characteristics for the management of patients undergoing treatment with dabigatran.

The international recommendations for the intake of VKA suggest that these low-risk interventions may be performed under ongoing anticoagulation in the therapeutic target range (INR 2.0–3.0) because some studies have demonstrated a high risk of thromboembolism when the therapy is discontinued, and the risk of severe bleeding is very low [16,17]. The same recommendation applies to treatment with LMWH (continuation of therapy without reduction of the dose) [18]. The risk of bleeding under dabigatran during these interventions is not known.

Based on extrapolation of the data obtained with conventional anticoagulants and the low risk of severe bleeding, the following procedure is recommended:

1. A preinterventional laboratory testing of dabigatran is not meaningful (**Recommendation 3C**).
  2. Interruption of treatment with dabigatran is not indicated (**Recommendation 3C**).

### Endoscopic high-risk interventions, organ puncture and high-risk vessel puncture

Common high-risk endoscopic interventions in the gastrointestinal tract include colonoscopy with polypectomy, ERCP with papillotomy, or endosonographic punctures. Many of these interventions are associated with a higher risk of bleeding (e.g., 0.07–1.7 % for removal of large bowel polyps) than low-risk interventions [18]. Due to the paucity of data, no distinction is made in the guidelines between removal of a pedicled polyp and a sessile polyp [16–18]. Organ punctures (liver, kidney, prostate gland, transbronchial biopsies, …), vessel punctures (e.g., when inserting a central-venous catheter without the possibility of compression from the outside, tunneled catheters or catheters of a large volume) bear a significant risk of bleeding, which may also involve the need for acute surgery [19].

The Summary of Product Characteristics provides no recommendation concerning the management of these interventions in patients undergoing treatment with dabigatran. The international recommendations for the intake of VKA support discontinuation of oral anticoagulation during these interventions, as in cases of major surgery [16–18].

The experts’ opinion is as follows:

1. A preinterventional laboratory testing of dabigatran is not meaningful (**Recommendation 3C**).
2. Dabigatran should be paused the day before the intervention (Day − 1;**Recommendation 1C**). The therapy should be discontinued earlier (at least 2 days) in case of renal failure (creatinine clearance < 50 ml/min;**Recommendation 1C**).
3. Treatment with dabigatran should be continued on the evening of the intervention, but no earlier than 4 h after the intervention (**Recommendation 1C**). In cases of a higher risk of bleeding due to the intervention and lower risk of thrombosis in the individual patient, one may consider delayed reinstitution of the therapy (24–72 h postinterventional;**Recommendation 1C**).

### Major surgery

Major surgical procedures are defined as those associated with a high risk of bleeding or the risk of severe complications in the event of bleeding. Typical examples of operations associated with a particularly high risk are mentioned in the Table 3. International guidelines for the management of patients with atrial fibrillation recommend interruption of oral anticoagulation during major surgery [9]. If the therapy is paused for a longer period of time and the risk of thromboembolism is high, LMWH therapy is recommended preoperatively. Postoperatively, depending on the type of surgery and the patient’s risk of bleeding and thrombosis, usually prophylactic anticoagulation is indicated to prevent venous thromboembolism. In Europe, dabigatran is approved for prevention of venous thromboembolism after elective knee and hip replacement surgery. The efficacy and safety (risk of bleeding) of dabigatran have been established only for these interventions. As the risk of bleeding is rather high during the first few days postsurgery, therapeutic anticoagulation should be reinstituted only after adequate hemostasis has been achieved (usually after 48–72 h).

**Table 3:** Operations or interventions with a high risk of bleeding or a high risk of severe complications in the event of bleeding. (Adapted from ACCP guidelines; [8])

Aortocoronary bypass operation
Cardiac valve surgery
Surgery for aneurysm of the aorta
Neurosurgical interventions or neuraxial anesthesia
Interventions performed close to the spinal cord
Major orthopedic surgery
Reconstructive plastic surgery
Tumor surgery
Urological operations in the prostate gland or bladder
Revision surgery for bleeding or infection

The Summary of Product Characteristics provides recommendations for the management of surgical interventions in patients with atrial fibrillation undergoing treatment with dabigatran [5]. These recommendations constitute the basis of the following expert recommendation:

1. A preoperative laboratory testing is usually not meaningful (**Recommendation 3C**). A dabigatran-sensitive coagulation test (Hemoclot test or ECT) may help to determine whether haemostasis is still impaired.
2. Dabigatran should be paused the day before the intervention (Day - 1;**Recommendation 1C**). The therapy should be discontinued 2 days in case of creatinine clearance 50–80 ml/min or interventions associated with a high risk of bleeding. The therapy should be paused 3–4 days before the intervention if creatinine clearance is < 50 ml/min (**Recommendation 1C**).
3. When the therapy is paused for longer than 1 day, in patients with atrial fibrillation and a CHADS_2_ score > 2 or those who have experienced an acute ischemic cerebrovascular syndrome the clinician should consider switching to weight-adapted LMWH. The switch should be made at the earliest 12 h (creatinine clearance > 50 ml/min) or 24 h (creatinine clearance < 50 ml/min) after the last dose of dabigatran (**Recommendation 1C**). According to the international recommendations, therapeutic doses of LMWH should be concluded at the latest 24 h before the intervention.
4. Postoperative prophylaxis of venous thrombosis should be administered in accordance with international recommendations (early mobilization, drug-based prophylaxis;**Recommendation 1A**)
5. Treatment with dabigatran at the usual dose (150 or 110 mg BID) should be continued from the third postoperative day onward when appropriate hemostasis has been achieved (**Recommendation 1C**). The drug used for prevention of thrombosis should be discontinued when the treatment with dabigatran is reinstituted (**Recommendation 1C**). In cases of prolonged risk of bleeding or high-risk patients, the clinician may consider delayed reinstitution of dabigatran therapy (**Recommendation 1C**).

Remark on 4: Dabigatran is only approved for prophylaxis of venous thromboembolism after elective hip or knee replacement surgery. If spinal or epidural anesthesia had been administered during this procedure the first dose of dabigatran (110 mg) should be administered no earlier than 2 h after removal of the catheter (Summary of Product Characteristics).

### Procedure in case of acute bleeding or acute operations

Studies concerning the management of acute interventions or bleedings do not exist [1]. The Summary of Product Characteristics contains few recommendations for the management of these interventions on the basis of the instructions of the RE-LY study protocol [5]. In addition to general measures for hemostasis such as compression, volume substitution, banked blood, etc., the following recommendation may be issued [20]:

1. Before an acute intervention, a diagnostic investigation of coagulation for the purpose of orientation—or if available a dabigatran-sensitive laboratory test—may help to estimate the magnitude of the existing anticoagulation in order to perform additional measures or postpone the intervention if necessary (**Recommendation 1C**). A normal thrombin time (TT) might rule out a significant effect of dabigatran but TT is often prolonged in clinical routine and especially with acute interventions due to other/preanalytical problems (e.g. blood sampling). A normal thrombin time (TT) might rule out a significant effect of dabigatran but TT is often prolonged in clinical routine and especially with acute interventions due to other/preanalytical problems (e.g. blood sampling). A normal aPTT does not rule out a significant effect of dabigatran.
  2. Postponing a therapeutic procedure (such as surgery) is not meaningful when the patient is in a life-threatening condition. However, if it would be possible to wait, the intervention should be performed not earlier than 12 h after the last dose of dabigatran (**Recommendation 1C**).
  3. Since dabigatran is eliminated predominantly by the renal route adequate diuresis must be maintained. As the plasma protein binding rate is low, one may consider hemodialysis in case of severe bleeding (62–68 % of the active substance can be hemodialyzed within 2–3 h;**Recommendation 1C**).
  4. In case of life-threatening bleeding, the following medications may be used: fresh-frozen plasma, prothrombin complex concentrate, FEIBA, or recombinant factor VIIa (**Recommendation 1C**).
  5. Platelet concentrates should be given in case of simultaneous thrombocytopenia or recent administration of platelet function inhibitors (**Recommendation 1C**).

#### Acknowledgement

Declarations of the authors about relations to industrial enterprises (statement of potential conflicts of interest)

Financial support for convening the consensus meeting including recompense for the authors was provided by Boehringer Ingelheim RCV GmbH & Co KG, Vienna, Austria. The company had no influence on the medical contents and recommendations of the consensus group. The consensus group convened by the coordinator (A. Weltermann) was multidisciplinary and was constituted in a representative manner for the circle of addressees (general practitioners, surgeons, anesthetists, and internists). Conflicts of interest among the individual participants are mentioned below.

#### Conflicts of interest

The following authors declare that no connections or financial or other conflicts of interest with third parties potentially interested in the contents of the guidelines exist:

Weltermann A, Brodmann M, Domanovits H, Eber B, Gottsauner-Wolf M, Halbmayer WM, Hiesmayr JM, Längle F, Roithinger FX, Watzke H, Windhager R, Wolf C, Zweiker R, Kyrle PA

The following authors declare that they have served as advisers, experts, lecturers, or worked in a scientific advisory board, or participated in studies for industrial enterprises or received financial support for performing research projects of industrial enterprises:

Weltermann A: Bayer Health Care, Boehringer Ingelheim, Sanofi-Aventis

Brodmann M: Abbot Vascular, Actelion, Bayer Health Care, Boehringer Ingelheim, Medtronic, Sanofi Aventis

Domanovits H: Bayer Health Care, Boehringer Ingelheim,http://www.bms.com/careers

Bristol-Myers Squibb, Sanofi Aventis, Pfizer

Eber B: Bayer Health Care, Boehringer Ingelheim

Gottsauner-Wolf M: Boehringer Ingelheim

Halbmayer WM: Boehringer Ingelheim

Hiesmayr JM: Baxter, Boehringer Ingelheim, Fresenius Kabi, Nestlé, Novo Nordisk

Kyrle PA: Bayer Health Care, Boehringer Ingelheim,http://www.bms.com/careers

Bristol-Myers Squibb,http://www.daiichi-sankyo.de/

Daiichi Sankyo, Sanofi-Aventis

Längle F: Boehringer Ingelheim

Roithinger FX: Astra Zeneca, Bayer Health Care, Boehringer Ingelheim,http://www.bms.com/careers

Bristol-Myers Squibb,http://www.daiichi-sankyo.de/

Daiichi Sankyo, Sanofi-Aventis

Watzke H: AOP, Astra-Zeneca, Baxter, Bayer, Boehringer Ingelheim, Cephalon, Grünenthal, Mundipharma, Pfizer, Roche, Sanofi-Aventis

Windhager R: Boehringer Ingelheim, Johnson & Johnson – DePuy, Stryker, Takeda

Wolf C: Boehringer Ingelheim

Zweiker R: AstraZeneca, Boehringer-Ingelheim, Lilly, Menarini, MSD, Novartis, Sanofi-Aventis, Servier, Takeda
